# The Caregiver Identity in Context: The Consequences of Identity Threat From Siblings

**DOI:** 10.1093/geroni/igaa057.1657

**Published:** 2020-12-16

**Authors:** Marissa Rurka, J Jill Suitor, Megan Gilligan

**Affiliations:** 1 Purdue University, Lafayette, Indiana, United States; 2 Purdue University, West Lafayette, Indiana, United States; 3 Iowa State University, Ames, Iowa, United States

## Abstract

Although siblings represent central members of the networks of caregivers’ and their parents, there has been limited attention to how siblings affect one another’s well-being during caregiving. In this paper, we draw from theories of identity and stress to examine the impact that siblings have on caregivers’ psychological well-being. Specifically, we employ a mixed-methods approach to explore whether caregivers’ perceptions that their siblings are critical of the care they provide their mother are associated with higher depressive symptoms, as well as the mechanisms underlying this association. Using quantitative data collected from 406 caregivers nested within 230 families as part of the Within-Family Differences Study, we conduct mediation analyses to examine whether perceived sibling criticisms affect caregivers’ depressive symptoms (a) directly, and/or (b) indirectly through sibling tension. We then analyze qualitative data collected from the same caregivers to gain insight into the processes underlying statistical associations. Quantitative analyses revealed that there was no direct relationship between perceived sibling criticisms and depressive symptoms; there was, however, an indirect relationship such that perceived sibling criticisms were associated with greater sibling tension, which in turn was associated with higher depressive symptoms. These quantitative findings were corroborated by qualitative analyses, which demonstrated that, in an effort to maintain their identity as a “good caregiver” despite sibling criticisms, caregivers often employed strategies that may have fueled tension in their sibling relationships. These findings highlight the importance of considering how the sibling networks in which caregivers are embedded shape their psychological well-being.

